# A conceptual review of the effectiveness of flipped learning in vocational learners’ cognitive skills and emotional states

**DOI:** 10.3389/fpsyg.2022.1039025

**Published:** 2023-01-13

**Authors:** Xiuqin Zhou

**Affiliations:** College Students' Mental Health Education Center, Shandong Management University, Jinan, China

**Keywords:** flipped learning, technology, vocational education, learner-centered approach, communicative competence

## Abstract

An inverted method of teaching is an instructional model where traditional classroom activities take place before class while class time is devoted to discussion, problem-solving, and interaction among students. Flipped learning is a learner-centered and technology-driven approach that benefits from the inverted method of teaching. Recently, instructors have begun to employ innovative pedagogies like flipped learning approach, to change the conventional practices in vocational education as flipped learning gives them a chance for professional development. In order to find out the reasons of the improvement of vocational education through the use of flipped learning approach, this review examined the effect of the flipped learning approach on vocational learners’ cognitive skills and emotional states in earlier studies. The earlier investigations showed the significant effect flipped learning approach on vocational learners’ emotions, such as engagement, motivation, self-efficacy, and their cognitive skills, including critical thinking, problem-solving, learning skill, learning strategies, and communicative competence. However, this review implicated that flipped learning, as a type of blended learning, may be beneficial for learners, instructors, and students’ parents to be aware of this valuable learner-centered approach in vocational education.

## Introduction

In recent years, educators have used the technological developments in education to create a more effective learning environment in which learning does not seem to be limited to the classroom environment and teachers may not be obliged to spend most of the class time delivering lectures; instead, they can have tutorial roles while students can also take different roles and be more actively involved in the learning process (Tan et al., 2017). One of the instructional models that follows this technology-related learning approach is flipped learning because it utilizes technological tools, including recorded lessons and videos, to create more engaging experiences for learners. The concept of flipped learning is that the teacher-student roles are changed in a way that the amount of direct instruction presented by the teacher during class time is minimized while the cooperative and collaborative contribution of students to the teaching process is maximized in class (Sams and Bergmann, 2013). Flipped classroom involves assigning what is traditionally done in the classroom as homework, and the homework is then completed in class; Instead of listening to a lecture in the class and doing homework at home, students watch video lectures and complete what has traditionally been known as homework in class under the guidance of the instructor (Baker, 2000). Flipped learning is a pedagogical approach that transforms direct instruction from the group learning space into a dynamic, interactive learning environment where the teacher guides students while applying concepts and engaging in the subject matter (MacKinnon, 2015; Teo et al., 2022). The goal of the flipped classroom is to maximize face-to-face time with students and instructional materials, which help increases students’ knowledge (Bull et al., 2012). The need for integrating technology in education as an innovation, motivates instructors and institutions, in the last years, to search for new educational methods that fit the needs of the current student profile (Al-Rahmi et al., 2021). A crucial stage of education is vocational education, which prepares some students for the labor market (Pambudi and Harjanto, 2020). Flipped Learning is particularly relevant in vocational education, which focuses on learning by doing and applying knowledge in a real setting (Hwang et al., 2015). In vocational learning, there is an even greater need for teachers and trainers to teach practice, rather than theory (Antonietti et al., 2022). According to CEDEFOP (European Centre for the Development of Vocational Training), vocational education is “the education and training which aims to equip people with knowledge, know-how, skills and/or competences required in particular occupations or more broadly on the labor market” (European Centre for the Development of Vocational Training (Cedefop), 2014). The flipped classroom approach, therefore, is favored in vocational education as it allows for valuable face-to-face time for practical applications and a more personalized experience for learners. Flipped learning can help educators by moving the theoretical content outside the classroom, and using class time for practical activities (Marshall and Kostka, 2020). Innovative models such as flipped learning can help improve the quality of vocational education, motivate students, and thus reduce the number of dropouts (Lai et al., 2020).

The flipped classroom approach offers the opportunity to move away from theory-based summative assessment methods to more practical activities and assessments based on developing students’ real-world skills (Fleischmann, 2021). Since, in vocational education, students learn by doing, the flipped classroom approach could give teachers more face-to-face time with their students to focus on work-orientated scenarios (Persky and McLaughlin, 2017).

Vocational education aims to cultivate students’ integrated ability to use English and to cultivate professional talents for society (Li, 2021). With the development of information technology, many new types of teaching models appear in the reformation, one of which is flipped classroom. Based on constructivism theory and modern information technology, flipped classroom teaching has gained more and more attention. This paper has researched the application of flipped classrooms in vocational education in order to provide relatively novel insights for teachers. Exploration in this field can help educators in many aspects of the classrooms to find new approaches to become more effective teachers and accordingly make a practical learning setting for increasing learners’ achievement in vocational contexts.

## Literature review

### The concept of flipped learning approach

Nowadays, technology is regarded as one of the most significant components of education. Based on von Lindeiner-Stráský et al. (2020), perspective, the growth of technology has radically changed instruction and education. They stated that the integration of technology into educational contexts, makes teachers re-evaluate their use of methodology to attain their objectives and improve learning effectiveness. Aiming to enhance learners’ achievements in educational contexts, the method known as a flipped classroom has drawn the attention of many researchers (e.g., Cheng P. W. et al., 2019; Jang and Kim, 2020; Tsai and Wu, 2020; Zou, 2020; Yulian, 2021). Durak (2018) declared that flipped learning approach, as a prominent approach, is highly useful in the integration of technology into education to increase success. Sajid et al. (2016) considered flipped learning one of the blended learning approaches. They asserted that blended learning is the combination of two instruction modes, e-learning and didactic (face-to-face) teaching. However, they maintained that blended learning is more traditional, while flipped learning is more digital. Collaborative and cooperative learning as two distinct methods can also be integrated into flipped learning approaches (Erbil, 2020). Utilization of the cooperative and collaborative learning methods in a flipped classroom environment is at a development stage, and there are no clear data regarding its results (Munir et al., 2018). However, the existing research has concluded that utilizing cooperative learning methods in a flipped classroom environment has a positive impact on students’ academic success levels (Zhang, 2018).

Flexible environment, learning culture, intentional content, and professional educator is regarded as the four pillars of flipped learning. Their purpose is to provide a practical roadmap for adopting the flipped learning approach (Sailsman, 2021). Hamden and McKnight (2013) stated that flipped classrooms allow a variety of learning modes; educators often physically rearrange their learning space to accommodate the lesson or unit, which might involve group work, independent study, research, performance, and evaluation. Having a flexible environment, the students do not feel tense and nervous and do not need to rush to get every detail in a compact lecture, rather based on the flexibility, the students feel free to get help from their peers or consult the teacher whenever they want (Demirel, 2016). Instead of being a passive object of teaching, the students are actively involved in their learning process and have the chance to participate in each step. Therefore, learners’ culture has been changed by the advent of flipped learning (Chivata and Oviedo, 2018). Hamden and McKnight (2013) pointed out that “educators use intentional content to maximize classroom time in order to adopt various methods of instruction such as active learning strategies, peer instruction, problem-based learning, or mastery, or Socratic methods, depending on grade level and subject matter” (p. 15). Professional educators as another pillar of flipped learning decide on the content, adapt the materials, choose the strategies, and maximize classroom interaction time (Bauer-Ramazani et al., 2016).

Agustini et al. (2021) pointed out that flipped learning is an appropriate approach to learning. In flipped learning approach, learners voluntarily and actively study the materials pre-class, and then other learning activities such as discussions, corporation, problem-solving, and practices are carried out during class time (Jung et al., 2018). Belmonte et al. (2019) stated that the pre-class self-learning phase brings the contents (previously prepared by the teachers) to learners’ private space, and learners can use web-based social media and technologies with the help of videos and related exercises out of the class. They pointed out that flipped learning approach encourages flexibility, both time (contents can be viewed as many times as necessary and at any occasion), and space (they can be viewed anywhere). They also asserted that in-class activities can be devoted to group activities during class time. Moreover, Rahman et al. (2020) pointed out that the flipped learning approach inverts teachers’ and students’ responsibilities in classrooms. Students are required to actively plan their learning process and interact with peers and teachers to acquire knowledge in the classroom.

Therefore, the shift of material consignment to the outside of the class and using the class time for higher-level activities like applying and examining the earlier learned materials are the primary components of flipped learning approach (Yilmaz and Baydas, 2017). Villalba et al. (2018) asserted that flipped learning approach, with its blended learning nature, shifts tasks traditionally executed in classrooms to external environments. Park et al. (2018) also compared passive instruction in traditional classrooms with flipped classrooms and highlighted the active role of students in the flipped classrooms as a student-centered participatory context. They also compared flipped classrooms and flipped learning. They mentioned that flipped classrooms create courses, texts, or lectures that can be viewed or read at the student’s pace, and flipped learning refers to the combination of in-class or face-to-face education with online learning.

Hinojo et al. (2019) stated that flipped learning approach turns the learner into an autonomous agent, who can significantly outperform observational, cognitive, and higher-order tasks. Based on Bloom’s revised Taxonomy, flipped learning approach provides an opportunity for learners to use active learning strategies both in and outside classroom (Jensen et al., 2015). Therefore, flipped learning approach is a pedagogical approach that encourages students’ active participation, promotes support from teachers, and peers to handle homework, and allows more free time in class (Guo, 2019). Flipped learning approach has been useful for different stakeholders, including learners, teachers, and parents. The following table includes some studies leading support on this issue.

**Table 1:** 

Authors	Title of the study	Methodology	Results
Doo (2021)	Understanding flipped learners’ perceptions, perceived usefulness, registration intention, and learning engagement	Survey-based	The advantages of flipped learning that have to do with online pre-learning sessions include facilitating understanding of lectures, providing review opportunities for learning, flexible learning time, and individualized learning. These advantages indicate that providing students with instructionally sound pre-learning sessions leads to successful flipped learning.
Bezzazi (2019)	The effect of flipped learning on EFL learners’ public speaking in Taiwan	Experimental	The results revealed that the flipped learning group significantly outperformed the conventional instruction group in the areas of body language and paralanguage. In addition, they did better in the areas of content and organization and developed other skills as will be detailed later. The findings can be an impetus for EFL instructors to adopt flipped learning in an English public-speaking course
Haghighi et al. (2019)	Impact of flipped classroom on EFL learners’ appropriate use of refusal: achievement, participation, perception	Experimental	The results of the questionnaires suggested that most participants of the flipped group enjoyed learning English in a flipped learning environment and willingly accepted Telegram as a suitable platform in learning the language. The authors present insights into the impact of flipped classrooms on appropriate use of English refusals, the participants’ perception of the flipped learning experience, their level of participation inside and outside the class, their impression of the online platform, Telegram, and offer implications for teachers to practice.
Shraideh et al. (2020)	Parents’ Perceptions of their Children’s achievement in the Reading Class Using a Flipped Classroom Model: A Case Study of Tenth Grade Female Students in Zarqa Directorate of Education.	Survey- based	Results showed that parents were positively influenced by their children’s feedback after experiencing each flipped course as well as their improved post-test scores. The majority of parents reported positive perceptions towards the implementation of the flipped classroom model and cited a wide-ranging list of merits.
Bond (2019)	Flipped learning and parent engagement in secondary schools: A South Australian case study	Survey-based	Parent involvement in and engagement with children’s learning in flipped learning has been shown to strongly influence student achievement, engagement, motivation and school completion.

Flipped Learning is based on a number of theoretical foundations. The first foundation is blended learning which transforms the lecture from class into online delivery and uses face-to-face class time (Abeysekera and Dawson, 2015). The second one is constructivism theory (Bruner, 1960), which indicates that learning occurs when a student works either with a more skilled adult or peer to solve problems that are just beyond her/his actual abilities (Jantakoon and Piriyasurawong, 2018). The core principles of constructivism are the followings: (1) learning is self-centered and self-directed; (2) learning is an active rather than passive endeavor; and (3) the instructor’s role is to foster critical reflection and facilitate the application and deeper understanding of new concepts (Aljohani, 2017). In constructivism, “knowledge is actively constructed by the learner, not passively received from the outside. Learning is something done by the learner, not something that is imposed on the learner” (Sjøberg, 2010, p. 3). The flipped learning approach supported by the constructivist theory should enable learners to engage in communicating, imaginative, and collaborative activities during knowledge construction (Kim and Bonk, 2006), and this approach requires learners to be active constructors of knowledge and use cooperative and collaborative learning, to reflect and, lastly, gain meaningful learning experiences in order to enhance their learning (Erbil, 2020). Vygotsky’s theory of mediation in digital learning is another theoretical construct of this review. Based on this theory, technology can be related to psychological and cognitive states. According to Zidoun et al. (2019), education programs should consider the role and impact of technological developments on learning. The concept of technological mediation, inspired by Vygotsky’s (1986) theory of tool mediation, aims to gain insight into the ways in which technology actively co-shapes the relation between people and the world through various mediating effects. De Boer et al. (2018) explain that this understanding of technological mediation emphasizes “the primacy of the relatedness between emotional states of people, technologies, and the world” (p. 300). And the last foundation is active learning (Lemmer, 2013), which emphasizes student activity and engagement in the learning process (Prince, 2004).

Recently, flipped learning has received a lot of attention in vocational and technical education. Flipped learning can help teachers and learners by moving the theoretical content outside the classroom, and using class time for practical activities. Innovative models such as flipped learning can help improve the quality of vocational education, motivate students, and thus reduce the number of dropouts.

### The notion of vocational education

Vocational education, as a type of education that highlights mastering skills to work, is to promote professional training and practically improve the skills of employees (Lai et al., 2020; Suharno et al., 2020). Vocational education is a combination of theory and practice in a balanced manner with an orientation to the readiness of its graduates. According to Papadakis et al. (2021), vocational education differs from general education mainly in terms of the focus on the dimension of the practical application of the knowledge provided to students. Vocational education was established against the background of the industry’s need for a professional workforce (Finlay et al., 1999). Billett (2011) stated that the aim of vocational education, from the traditional perspective, was to prepare learners for working. However, this aim becomes broader nowadays. He maintained that vocational education is one of the educational institutions that have a significant impact on the enhancement of human resources.

Djohar (2007) stated that from a school perspective, vocational education teaches people how to work effectively. An individual, learning how to work, will acquire vocational education, both at the secondary and post-secondary levels. As its ultimate goal, vocational education helps the students work in certain fields, and master their field competencies in the world of work following the education (Daly and Lewis, 2020). Another objective of vocational education is to reduce unemployment by equipping the graduates so they can compete with the provision of hard skills and soft skills (Tandirerung and Vitalocca, 2017). Higher vocational and technical teachers in vocational education are mainly to cultivate professional learners needed for vocational education (Ye et al., 2022). Nonetheless, vocational high school learners are needed to attend internships before they graduate from school despite the fact that the learning progression through firms is characterized by uncertainties that can undermine goal pursuit and subsequent attainment (Hong et al., 2021).

The need for the development of vocational learning concepts, in the era of the knowledge-based working world, is very urgent and essential (Utami, 2018). Suharno et al. (2018) asserted that the proper implementation of vocational education results in industrial development in a country. According to him, vocational learning is to develop basic competencies, indicators of competency achievement, learning objectives, learning media, learning methods, and learning strategies. He also declared that establishing these schools can lead to the welfare of the local community. He declared that a country should pay more attention to the development of vocational education, and the stakeholders should realize the nature of this education. Therefore, it is accountable for the quality of training experts in numerous fields (Belovitskay et al., 2021).

Based on Widiatna (2019), the domain of the learning process, especially in vocational education, must include cognitive (knowledge), psychomotor (skills), and affective (attitude). Many vocational high school teachers currently use traditional approaches of instruction, in which the role of teachers is significant, and students are only good listeners in the classroom (Basori, 2018). However, by the emergence of technologies, instruction has been positively affected (Rabiman et al., 2020). In the current digital era, technology is very significant in the growth of vocational education (Krismadinata et al., 2020). Teachers, involved in blended learning processes in vocational education, tend to adopt different approaches to teaching and learning designs (Bliuc et al., 2012). The learning approach in the widely used blended learning environment is flipped learning (Thai et al., 2017). In addition to accommodating in a blended learning environment, flipped classroom has an impact on better student learning outcomes (Hao and Lee, 2016).

### The role of flipped learning approach in vocational education

The flipped learning approach is a form of learning approach that can apply in vocational education. In order to examine the effect of flipped learning in job-based education terms of learners’ cognitive and emotional states, this conceptual review scrutinizes some recent studies on this issue. Some studies have been done on the effect of flipped learning on job-based learners’ cognitive skills and achievements. Bhagat et al. (2016) investigated higher vocational learners’ learning mathematics concepts in a flipped classroom. The quasi-experiment results indicated that learners who used the flipped classroom approach had higher mathematics achievements than the control group, which used a conventional teaching approach. In addition, low-achieving students in flipped classrooms had higher mathematics achievement scores than students in the control groups. They argued that flipped learning approach can engage in higher-order thinking activities. Singh et al. (2017) studied the effect of flipped learning on Technical and Vocational Education and Training (TVET) students. They found that flipped learning enriched learning environment with well-planned learning lesson plans. Park (2018) studied the effect of flipped classrooms on vocational learners in the field of radiology. They found that knowledge, skills, and attitudes in flipped classrooms improved. Moreover, they found that vocational learners improved their responsibility, problem-solving ability, creative thinking, cooperative ability, and communication ability through flipped learning approach. Dong (2018), in his study, revealed that flipped learning approach enables economic and management learners in vocational education to advance in studying and practicing. He argued that integrating the project-type teaching of the flipped classroom requires learners to take proactive measures before class and actively devote themselves to the learning of new knowledge. If vocational learners have an incomprehensible problem in their course, flipped classrooms provide opportunities for learners to record their problem and discuss it with their classmates or teachers in class. He also found that flipped classrooms have special designs, in which deeply link between learners and the working process occurs, and flipped classrooms provide learners a context for applying theory to practice. Bahramnejad Jouryabi (2019) investigated the effectiveness of flipped classroom model on lower-level and higher-level groups of students’ academic achievement in a vocational high school. Their study indicated that higher-level learners outperformed compared to their lower level counterparts. They indicated that learners’ level of proficiency is considered an influencing factor in the successful implementing of EFL flipped classrooms. Chen and Hwang (2020) explored the influence of concept mapping-based flipped learning on vocational learners’ listening-speaking strategy, learning achievement, and their critical thinking awareness. They found that concept mapping-based flipped learning has a positive and significant influence on EFL learners’ English speaking performance and critical thinking awareness. Yorganci (2020) investigated the effect of E-learning, blended learning, and flipped learning approaches on mathematics achievement. He found that the mathematics achievement of students in flipped classroom was significantly higher than those of the students of E-learning and blended learning. Montaner-Villalba (2021) examined how students, in tertiary education, perceived ESP academic writing skills within the field of Business English in flipped classrooms. Using a questionnaire and a focus group interview, he proved that students’ perceptions towards academic ESP written competence, using Business English, within the Flipped Learning approach was positive.

Some studies have been done on the effect of flipped learning on vocational learners’ emotional states. Xin-yue (2016) explored the motivation of Chinese vocational learners in flipped classrooms. His study revealed that the flipped classroom approach does stimulate students to invest more time and effort prior to instruction and during class learners do participate in communicative language exercises more enthusiastically. He recommended the incorporation of periodic rotation within the class, addition of certain teacher-led instruction, and informal evaluation with group members to help further improve the teaching/learning outcome of the flipped classroom approach. States. Lai et al. (2020) examined the impact of team-based flipped learning classes on vocational high school learners’ learning achievement and motivation who majored in business management. They used team-based groups in flipped classrooms, which require collaboration, and they focus more on the time management and quality control throughout the learning task. Students were required to watch the 30-min videos on their own time before class. When the class began, students had 20 min to apply the knowledge learned in the lecture videos. However, in the control group, the didactic method with small group discussion was used. Both experimental and control groups took the achievement pre-test and the revised Motivational Strategies Learning Questionnaire (MSLQ) 1 week before the experiment. Students were required to complete the post-test and the same MSLQ immediately after the experiment. The findings of the study showed that using flipped learning approach significantly affects vocational high school learners’ learning performance and motivation. The vocational high school learners in the flipped classes had better discussion quality than those in the traditional groups. In other words, vocational learners in the team-based flipped classrooms presented more from economics perspectives and analyzed the questions systematically. In terms of vocational learners’ performance, Cheng L. et al. (2019) undertook a literature review to evaluate the overall effect of the flipped classroom on student learning outcomes. This meta-analysis compared flipped classrooms with traditional classrooms, and they found the results were significantly in favor of the flipped classroom approach.

Yu et al. (2019), studying nursing students, suggested that the flipped classroom is more effective for the nursing students’ skill competence than traditional teaching in China. Li et al. (2020) compared the effect of the flipped classroom using massive open online course (MOOC) and lecture-based learning on Chinese nursing students’ theoretical scores. In MOOC-based flipped classrooms, students were required to choose proper video for self-learning toward the predominant objective of developing their learning effectiveness. They found that nursing students outperformed in MOOC-based-flipped classroom. They justified their results by arguing that flipped classrooms stimulate active learning. Moreover, their study revealed that flipped classrooms provide learners the opportunities to understand content at their own pace while perusing online materials. They also asserted that classroom tasks are intended to allow students to focus on applying the content to better understand the materials being taught. These activities can be completed individually or in peer teams, thus shifting the teacher’s role from the source of knowledge to the promoter of student learning. Furthermore, they argued that in flipped approach, class time is allocated to students to use, analyze, and assess their knowledge. Therefore, the flipped classrooms can enhance students’ motivation, satisfaction, academic performance, and engagement in vocational education.

Yorganci (2020) compared the effect of flipped learning, E-learning, and blended learning approaches on the vocational learners’ performance, self-regulation, and self-efficacy majoring in mathematics. He employed Mathematics Achievement Test, Barnard et al.’s (2009) Online Self-Regulated Learning Questionnaire, and Umay’s (2001) Mathematics Self-Efficacy Scale in his study. His findings showed that flipped learning approach significantly increases learners’ self-regulation, performance, and self-efficacy. He argued that the increased sense of competence of students in flipped learning classes inspires them to solve more demanding problems, and it is an essential factor in math performance due to the convenience of online learning resources at any time for supplementary tasks. He also maintained that web-based courses in flipped learning settings give more opportunities to students to deal with high-level cognitive procedures and to process the information they acquired. Regarding the effect of flipped learning on self-efficacy, he stated that flipped learning approach increases learners’ self-efficacy, which boosts vocational learners’ effort in their actions. He stated that the development of self-efficacy, in flipped classrooms, can improve vocational learners’ behavior, since learners can better use cognitive strategies to have effective learning. He also justified the effect of flipped learning approach on vocational learners’ self-regulation by arguing that flipped learning approach activates learners in their vocational education, allows them to regulate their acquired experiences in multiple ways, and creates learning, and provides them an opportunity to obtain a strong learning mechanism in which they can put the acquired information into real life by reiterating and they can monitor their thinking process. His study implicated that the usage of flipped learning approach in mathematics courses has a positive effect on the learning process of students.

Lo et al. (2021) investigated the effect of flipped and traditional learning approaches on vocational high school students’ learning achievement and learning strategies. The participants of their study were learners majored in electrical engineering, and they employed learning strategy scale of students in vocational high schools as the instrument. Moreover, the students’ scores on the Testing Center for Technological and Vocational Education Test were used to evaluate their learning effectiveness. Their study showed the effectiveness of flipped learning approach in fostering learners’ learning achievement in the electronics course. They also indicated that flipped classrooms enable vocational high school learners to further improve their learning strategies, including learning motivations, self-evaluation, and problem solving. They argued that learners with lower scores in flipped classrooms can simply ask their peers for information on notions they did not comprehend, whereas, learners, with higher scores, found teaching peers helpful for their learning, and this help them review and elucidate their notions. Therefore, discussions in the flipped classrooms enhance learners’ problem-solving. Their study also showed that flipped learning approach can provide the foundation of learners’ self-regulation learning, which fosters learners’ motivation and allows learners to follow a better academic performance. They also found that flipped classrooms can improve learners’ achievement in their performance in long-term training.

Belovitskay et al. (2021) also declared that the application of the flipped learning approach can improve the forms and methodology of the educational activity to foster vocational education and develop future competitive professionals in the field of agriculture. Vocational learners may, either independently or through direct interaction with the teacher in the classroom, or with the use of e-learning and distance learning technologies, determine the topics to learn. They stated that flipped learning approach creates conditions for learners to use and manage the information effectively. Moreover, they pointed out that flipped learning approach offers professional identification by future specialists. However, the use of flipped learning approach in vocational education helps instructors to monitor the flipped classes and learners’ self-control, and to evaluate learning outcomes by establishing educational activities with face-to-face and e-learning technologies. They mentioned that flipped learning approach provides more interaction and more feedback in face-to-face sessions among learners. They also asserted that flipped classrooms provide opportunities for instructors to store the results of the educational process in electronic form. Their study also showed that flipped learning approach in vocational education provides educational conditions for the growth of learners’ critical and flexible thinking, and inspires them to seek knowledge and educational material. Papadakis et al. (2021), in their action research, found that using the flipped approach as a blended learning model within the classroom with the use of technology, can solve some drawbacks of the traditional educational process to improve the quality of teaching and facilitate of learning in vocational education. Using LAMS as a platform in flipped classrooms, they found that integrating flipped learning in vocational education can provide diversity, skills, and abilities among vocational learners and can equip them with the necessary knowledge for their professional rehabilitation. They asserted that flipped classrooms offer reusable sequences of learning activities through LAMS, with an observed enhancement of their active involvement in the learning process. They also maintained that flipped classrooms strengthen the interaction and communication community between the students and teacher, and promote collaboration between team members. Flipped learning approach enables the operative management of teaching time within the classroom due to the reversal of the educational process. Jularlark et al. (2021) found that learning management in flipped educational contexts improves learner engagement and enhances learner knowledge in vocational education. Their study revealed that brainstorming and collaboration on creating a workpiece are the advantages of using the flipped approach in vocational education. Al Mamun et al. (2022), in their study, revealed that flipped learning approach can deal the challenges of complex educational applications in different fields of engineering education.

## Discussion

To be able to keep up with the rapid advances in technology, the subsequent change in the instructional methodologies, and at the same time to hold the learners’ attention who are bored with the traditional book, and pencil classes, teachers have to update themselves, and use the latest accessible approaches. The flipped learning approach, as one of the pedagogical approaches, can be employed to advance learners’ performance. This review delved into the effect of flipped learning approach on vocational education. The earlier studies showed that flipped learning approach can enhance learners’ positive emotions such as motivation (Yu et al., 2019; Lai et al., 2020). The studies revealed that learners are motivated to do in-class discussions in flipped classrooms. This upsurge in student motivation could be a result of student satisfaction with their experiences of flipped learning. Moreover, the previous literature indicated the significant and positive effect of flipped learning on learners’ level of self-efficacy and emotion regulation (Yorganci, 2020; Fan and Wang, 2022). It can be concluded that flipped learning can provide the students with more than expected opportunities for success. The studies on self-efficacy and flipped classes highlighted positive attitudes because of the satisfaction derived from meeting basic cognitive needs such as a sense of competence, autonomy and social interaction (Ha et al., 2019). This could result in an increase in self-efficacy, especially in technology-integrated classes where students are claimed to have become autonomous, self-regulated and self-confident through participation and interactions in a technology-enhanced learning environment (Yang, 2017; Namaziandost et al., 2018; Han and Wang, 2021). Moreover, flipped learning can improve learner engagement (Yu et al., 2019; Jularlark et al., 2021). Positive collaboration, as well as peer teaching and learning, were particularly encouraged through the flipped approach, as were increased enjoyment, participation, and improved student-teacher relationships (Xie and Derakhshan, 2021). Moreover, vocational learners’ cognitive skills, including critical thinking (Bhagat et al., 2016; Belovitskay et al., 2021), problem-solving (Park, 2018; Lo et al., 2021), creative thinking (Park, 2018), learning skill (Cheng P. W. et al., 2019; Lai et al., 2020), learning strategies (Lo et al., 2021), and communicative knowledge (Park, 2018; Belovitskay et al., 2021) can be influenced by flipped learning. Earlier studies showed that flipped learning is a present and future learning that needs to be mastered by the teaching staff, therefore it is necessary to develop learning activities related to flipped classrooms.

### Implications

This review has some pedagogical implications for learners, teachers, and students’ parents. Learners can take advantage of the current review in various ways. For example, they can identify their learning strategies, and act based on their strategies, in accordance with flipped classrooms. They can ask instructors to provide materials that they like and matches their learning strategies in flipped classrooms in order to increase engagement, creative thinking, motivation, and critical thinking. At the same time, they can practice extending their preferences to be able to take more advantage of the presented materials.

Vocational educators and teachers can develop new and customized ways to foster the flipped model effectiveness in their teaching context, and they can modify it based on teachers’ and students’ needs. A flipped classroom may bring many benefits for teachers. It helps them to quit the traditional ways of teaching, and effectively apply the new approaches to teaching. Technology can free up the teacher to move towards a non-synchronous student-centered learning environment where each student receives an individualized education program.

Vocational teachers are supposed to be aware of the rules and regulations of flipped learning approach. This awareness can help teachers to use effective strategies in order to use this teaching approach. They need to develop digital expertise to provide immediate feedback, adequate guidance, strong support throughout the flipped instruction, and to build interconnectivity between pre-class materials and in-class tasks, based on the flipped approach. Indeed, teachers who are not trained in constructivist approaches may not be interested in utilizing flipped learning approach in their teaching programs. Therefore, it is recommended that the administrators provide training courses for teachers to acknowledge the flipped classroom paradigms, and help them to use this approach with confidence. Moreover, teachers should be given ample opportunities to observe some experienced teachers who often flip their instruction. Also, administrators should be aware of educational needs, and try to support these needs in any way possible. Secondly, employing flipped learning approach requires time optimization, and it is best done gradually. For this purpose, teachers can flip a few lessons at first steps, and then try to cover a term or entire academic year. They should know how to optimize time to develop activities, especially student-centered activities, instead of spending their time lecturing. Teachers can help students to deal with various learning activities, so they will reach stability. It will be recommended for teachers to use flipped learning approach to help students become self-aware of how they deal with classroom activities, and understand the main target of the activity. The teachers should teach students to be self-aware in order to understand the difficulties they encounter.

In flipped classrooms, teachers can use remote collaborative learning activities to make a little more thought and preparation than in-person ones, but they are equally rewarding. Moreover, they can use classroom debates, and they can appoint learners to represent two sides of a timely or controversial issue, and have them present arguments defending their position. In addition, they can employ breakout discussions, and have students discuss a question, issue, or problem. At the end of the session, they can have each group report on their conclusions. They can also use jigsaw activities by breaking the class up into groups of four or five students. They can have each group member research a different issue or component of the broader subject. During class time, they can have them come together and share their findings. They are recommended to employ seminars in their flipped classrooms, and they can get students to take turns leading a class discussion on a topic they have researched. Moreover, to reduce learners’ workload, sufficient time should be given to learners in the pre-class phase, whereas learning strategies, and time-management training should be provided to maximize learners’ time use. Teachers can manage the class time and learner engagement regularly to arouse motivation. They are required to decrease learners’ anxiety and disengagement, and they need to increase learners’ motivation irrespective of educational problems in vocational contexts to enrich learner skills. They should talk to learners about their internal, and external motivation in online contexts to be aware of learners’ personality traits which help them to engage enthusiastically in flipped learning contexts.

### Suggestions for further research

Further research could find how technological advances could make flipped classroom experiences more challenging and engaging in vocational education. Some positive emotional constructs, such as learner enjoyment, well-being, pedagogical love, resilience, and grit (Wang et al., 2021), are suggested to be investigated among learners in vocational education. Moreover, the effect of flipped learning approach on these emotional construct is required to be examined in contexts like vocational education. Future research is also needed to find the effects of well-structured flipped classes versus ill-structured ones on learners’ learning, using various educational designs and strategies. Future research should be devoted to the effect of gender, socio-economic background, age on vocational learners’ academic achievement and emotional states in flipped classrooms. Furthermore, the effect of flipped learning approach on the proficiency level of vocational learners in different academic fields should be highlighted for the future. In addition, conducting case and phenomenological investigations, provides a good starting point for a discussion on the reasons behind the effectiveness of flipped learning in vocational learners’ cognitive skills and affective states. Finally, it is helpful to see more research on the teacher’s role in the classroom when the class is flipped. All of these ideas for further research would be good ways to extend our understanding of flip classrooms in vocational education.

## Author contributions

The author confirms being the sole contributor of this work and has approved it for publication.

## Conflict of interest

The author declares that the research was conducted in the absence of any commercial or financial relationships that could be construed as a potential conflict of interest.

## Publisher’s note

All claims expressed in this article are solely those of the authors and do not necessarily represent those of their affiliated organizations, or those of the publisher, the editors and the reviewers. Any product that may be evaluated in this article, or claim that may be made by its manufacturer, is not guaranteed or endorsed by the publisher.
